# Heterotopic pancreatic tissue in the gall bladder neck and lymph node surrounding cystic duct identified during cholecystectomy for chronic calculous cholecystitis: a rare case report from Syria

**DOI:** 10.1093/omcr/omac068

**Published:** 2022-06-23

**Authors:** Moatasem Hussein Al-janabi, Leen Ismail, Marah Yousef Wassouf, Jamal Sulaiman, Rabab Salloum, Zuheir Al-shehabi

**Affiliations:** Department of Pathology, Cancer Research Center, Tishreen University Hospital, Lattakia, Syria; Tishreen University Faculty of Medicine, Lattakia, Syria; Tishreen University Faculty of Medicine, Lattakia, Syria; Department of General Surgery, Tishreen University Hospital, Lattakia, Syria; Department of Pathology, Tishreen University Hospital, Lattakia, Syria; Department of Pathology, Director of Cancer Research Center, Tishreen University Hospital, Lattakia, Syria

## Abstract

Heterotopic pancreas is a congenital anomaly defined as the presence of the pancreatic tissue outside its normal location, which is usually discovered incidentally. We describe a rare case of heterotopic pancreatic tissue in the neck and the node surrounding the cystic duct of the gallbladder. A 33-year-old female presented with right upper quadrant abdominal pain aggravated after fat meals. The diagnosis was made as chronic cholecystitis. Laparoscopic cholecystectomy was performed. Pathologic examination showed a lobulated nodule connected to the cystic duct. Microscopically, this node consisted of all components of pancreatic tissue. Localization of heterotopic pancreatic tissue in the gallbladder is exceedingly rare. Less than 40 cases of heterotopic pancreas in the gallbladder have been reported worldwide. The ectopic pancreas is an extraordinary congenital entity. Although pancreatic tissue in the lymph node is an exceptional finding, pathologists should be aware of it because it may be confused with tumor or metastasis.

## INTRODUCTION

Heterotopic pancreas (HP), also referred to as an ectopic pancreas, pancreatic rest and accessory pancreatic tissue is defined as the congenital presence of pancreatic tissue in an anatomic place with no communication with the main pancreas [1]. The most common sites of HP are the stomach, duodenum, colon and Meckel diverticulum. It can also be found uncommonly in the gallbladder, biliary tract, spleen, liver, mesentery, lung and pelvis [2]. The gallbladder is a very rare location that is involved by pancreatic rest [3]. The ectopic pancreas is usually an incidental and asymptomatic finding, but it may be present with non-specific gastrointestinal symptoms. In this report, we present a case of HP in the neck of the gallbladder and around its cystic duct.

## CASE REPORT

**Figure 1:**
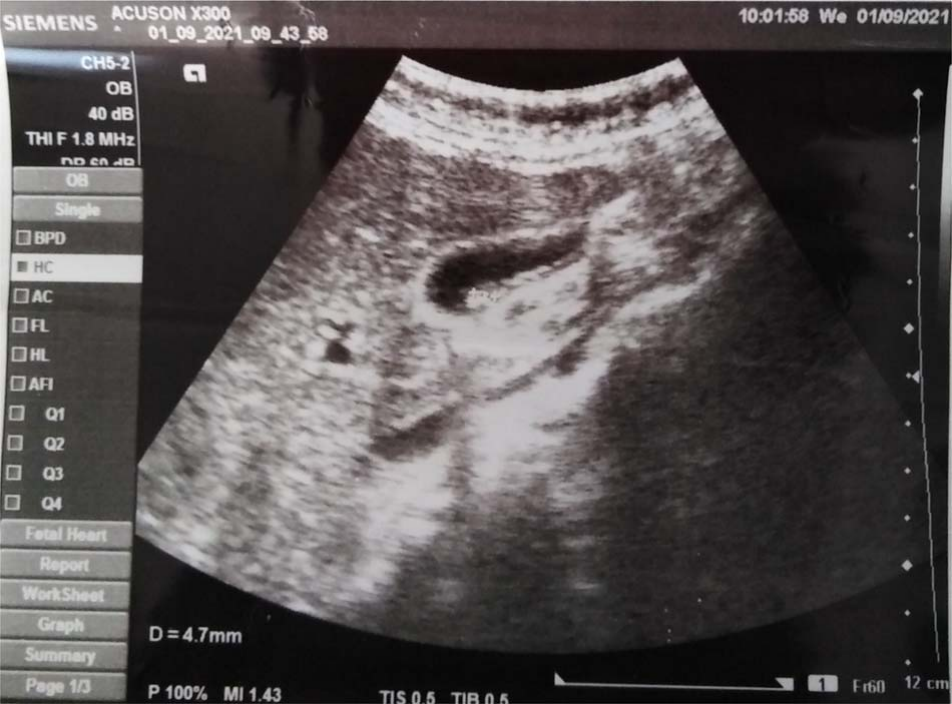
An ultrasonographic image of the gallbladder showed wall thickening with multiple gallstones in the lumen.

**Figure 2:**
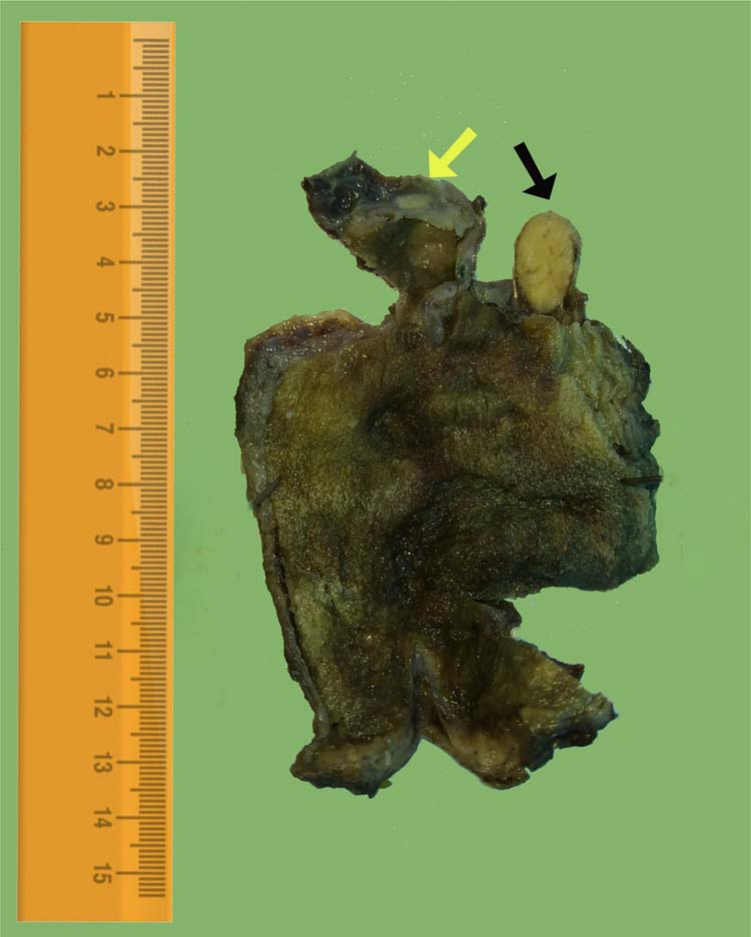
Gross image of the excised gallbladder, cut section revealed a well-circumscribed, yellow nodule in the area of the cystic duct (black arrow) and a similar small nodule in the neck region of the gallbladder (yellow arrow).

**Figure 3:**
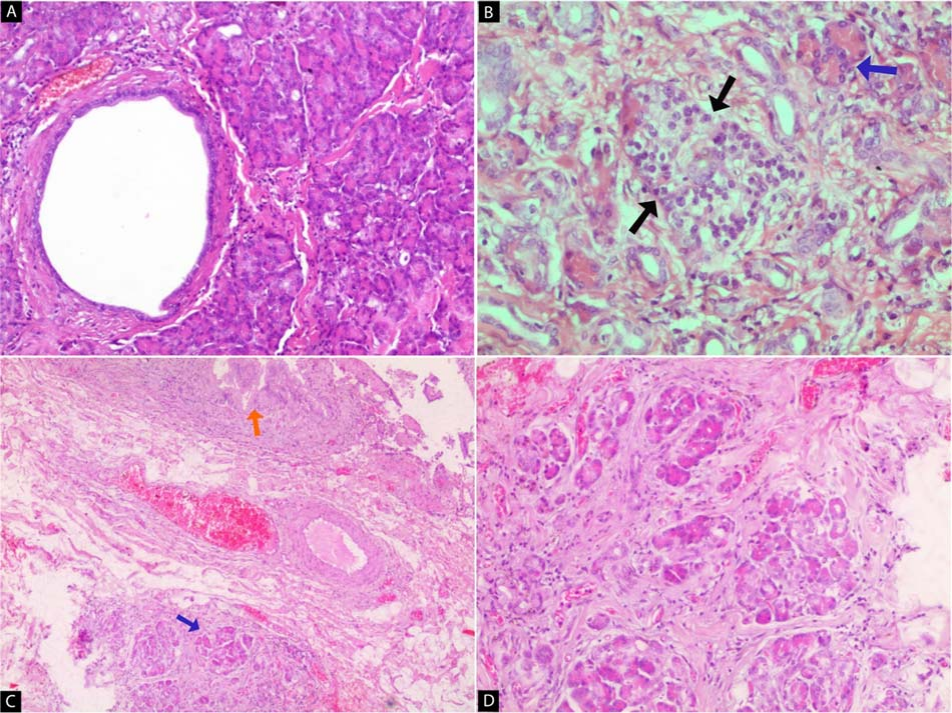
Microscopic images of the gallbladder. (**A**) Presence of pancreatic tissue; large duct and many acini (H&E ×100). (**B**) The islet of Langerhans (black arrows) with exocrine acini (blue arrow) (H&E ×200). (**C**) The mucosa of the gallbladder (orange arrow) and pancreatic tissue in the serosa (blue arrow) (H&E ×40). (**D**) A large number of acini in the serosa (H&E ×100).

A 33-year-old female presented to the emergency department at Tishreen University Hospital in 2021 with repeated attacks of right upper quadrant abdominal pain radiating to the back and right shoulder and worsened by fatty meals. These symptoms persisted for 2 months before her admission to our hospital. The patient was a non-smoker and non-alcoholic. She had no previous surgery or allergy to any drugs. On physical examination, there was tenderness in the right upper quadrant with a positive Murphy’s sign. Laboratory tests showed hemoglobin of 12.4 g/dL, WBC of 5.1 mm^3^, serum amylase of 74 U/L (40–140) and blood glucose of 85 mg/dL. Her liver and renal function tests revealed no abnormality. Abdomen ultrasound showed gallbladder wall thickening and multiple gallstones in the lumen, the largest was 4.7 mm in diameter (Fig. 1). Depending on the diagnosis of chronic calculous cholecystitis, laparoscopic cholecystectomy was done. On pathological examination, the gallbladder measured 7 cm in length and 3 cm in diameter with a wall thickness $\sim$of 0.5 cm. The serosa was unremarkable. Gross inspection of the excised gallbladder showed a firm mass surrounding the cystic duct measuring 1.2 cm. After opening the specimen, another mass was found in the serosa of the neck region about 0.5 cm in diameter. Cut sections revealed well-circumscribed, yellow and lobulated nodules (Fig. 2). The mucosa was intact, green and velvety. The H&E-stained sections showed an ectopic pancreatic tissue in the large mass, composed of lobules of exocrine pancreatic acini, ducts (Fig. 3A) and islets of Langerhans (Fig. 3B). On the other hand, only acini were seen in the serosa of the gallbladder (Fig. 3C and D). The apical area of acinar cells contained eosinophilic granules, whereas the basal region is markedly basophilic. Residual lymphatic tissue in the lymph node was not seen. The postoperative period was uneventful and the patient was discharged on the same day, without any complications.

## DISCUSSION

HP is an infrequent congenital entity, defined as pancreatic tissue that lacks anatomical or vascular communication with the true pancreas [4]. HP can be occurred anywhere in the gastrointestinal tract. The most frequent locations are the stomach, duodenum and jejunum [2, 3]. By contrast, the presence of ectopic pancreas in the esophagus, gallbladder, mesentery, spleen, mediastinum or fallopian tubes is rare [2, 3]. However, the gallbladder is an extremely rare site for the HP [3]. The first reported case of HP in the gallbladder dates back to 1916 [5]. Until the present study, >40 cases of HP in the gallbladder have been reported worldwide [6]. In most patients, this condition is an incidental finding on upper endoscopy or laparotomy and it is generally asymptomatic. However, it may be complicated by inflammation, bleeding, obstruction or malignant transformation [7]. The differential diagnosis of the enlargement of cystic duct lymph node includes cholecystitis, tuberculosis, ectopic tissue and lymphoma or metastasis [8]. Our first diagnosis was reactive lymphadenopathy in context of chronic cholecystitis. The microscope examination showed ectopic pancreatic tissue with no residual lymphatic tissue. Several theories have been proposed to explain the pathogenesis of HP; the most widely accepted is that the tissue is separated from the pancreas gland during the rotation of the gastrointestinal tract during embryonic life [5]. The literature reported one case of HP in four lymph nodes around the common hepatic artery. The pathogenesis of HP in the lymph node is unclear. Two possibilities have been proposed; a failure during embryologic development or lymphatic migration of epithelial elements [2]. Ectopic pancreatic tissue is usually located in the neck or fundus of the gallbladder [9]. Preoperative diagnosis is rarely clinically or radiologically possible because it is a very rare pathological entity, which diagnosis is confirmed by microscopic examination [9]. In our case, in addition to the neck, it has also been found in the cystic duct lymph node, which is considered a very exceptional site. Histologically, the ectopic pancreas can be complete heterotopia, similar to the normal pancreas, composed of exocrine acini, duct and islets of Langerhans, or incomplete heterotopia if the endocrine elements were absent. Based on microscopic findings, HP has been classified into three types by von Heinrich in 1909. Type 1: all components of pancreatic tissue (acini, ducts and islets of Langerhans) are present; Type 2: contains only a few acini and ducts, but lacks endocrine elements; Type 3: only proliferating excretory ducts with the absence of acini and islets cells [10]. According to what was mentioned above, our case was considered to be complete heterotopia and Type 1 of the Heinrich classification.

## CONCLUSION

Pancreatic heterotopia is an exceptional event especially when it is localized to the gallbladder. Although HP in the lymph node is unusual, pathologists should be aware of it because it may mimic tumor or metastasis.

## CONFLICT OF INTEREST STATEMENT

The authors have no conflicts of interest to declare.

## FUNDING

No funding.

## ETHICAL APPROVAL

No ethical approval is required for this case report.

## CONSENT

Written consent was obtained.

## GUARANTOR

Rabab Salloum.

## Supplementary Material

Revised_omac068
